# Variability of organelle genomes in a collection of early maturing soybean varieties

**DOI:** 10.18699/vjgb-26-23

**Published:** 2026-04

**Authors:** V.V. Aleksandrovich, M.G. Siniauskaya, A.P. Shatarnov, O.G. Davydenko

**Affiliations:** The Institute of Genetics and Cytology of the National Academy of Sciences of Belarus, Minsk, Belarus; The Institute of Genetics and Cytology of the National Academy of Sciences of Belarus, Minsk, Belarus; The Institute of Genetics and Cytology of the National Academy of Sciences of Belarus, Minsk, Belarus; Soya-North Co Ltd, Kolodishchi, Minsk oblast, Belarus

**Keywords:** soybean, Glycine max, genetic variability, organelles, chloroplasts, mitochondria, NGS, соя, Glycine max, генетическая изменчивость, органеллы, хлоропласты, митохондрии, NGS

## Abstract

Variability of the genomes of cellular organelles (chloroplast and mitochondria) is an important component of the overall variability of the plant genome. A large amount of data has already been obtained on the comparative characteristics of the organization of organelle DNA sequences for different groups of plants. This paper presents new original data on the variability of mitochondrial and chloroplast genomes in soybean (Glycine max (L.) Merr.), a crop of great economic importance widely cultivated in Central Europe, including the Republic of Belarus. Initially, we supposed that the peculiarities of soybean organelle DNA sequence or organization promote certain soybean cultivars to be the best maternal and others, alternatively, the best paternal forms. As a result of the study, new complete nucleotide sequences of chloroplast and mitochondrial genomes of 46 soybean samples were obtained by the next generation sequencing method (NGS) on the Illumina platform. A comprehensive bioinformatic comparative study of intraspecific organelle genome variability in 46 soybean varieties of diverse geographical origin was conducted. Polymorphic loci of genomes were discovered. Data on DNA variability were verified by Sanger sequencing. The spectrum of organelle DNA variability of cultivated soybean was represented by three chloroplast DNA haplotypes (C1–C3) and five mitochondrial DNA haplotypes (M1–M5). A comparatively low level of intraspecific variability of organelle genomes in G. max was revealed. The soybean chloroplast genome had a lower level of sequence variability than the mitochondrial genome. A set of DNA markers for polymorphic loci of organelle genomes was developed, allowing the differentiation of varieties of the studied group into plasmatypes. Additionally, 90 soybean samples from the collection were studied using PCR followed by Sanger sequencing. The low level of intraspecific variability of organelle genomes in G. max was confirmed on the extended group of samples. The majority of cultivars were represented by three plasmatypes – C1/M1, C2/ M2 and C1/M3. 46 complete chloroplast DNA sequences have been deposited in NCBI GenBank. The hypothesis that organelle DNA influences the combining ability of different varieties has not yet been confirmed. A more detailed study of the mechanisms of nuclear-cytoplasmic interaction is required, as well as a search for nuclear markers that affect the expression of organelle genes.

## Abstract (ru)

Изменчивость геномов клеточных органелл – хлоропластов и митохондрий – является немаловажной компонентой общей изменчивости генома растений. Получено большое количество данных о сравнительных особенностях организации последовательностей органельных ДНК для различных групп растений. В настоящей работе представлены новые оригинальные данные об изменчивости геномов митохондрий и хлоропластов у сои (Glycine max (L.) Merr.), важной хозяйственной и пищевой культуры, широко возделываемой на территории Центральной Европы, в том числе и в Республике Беларусь. Рабочей гипотезой нашего исследования изначально стало предположение: возможно, особенности изменчивости последовательности или структуры ДНК органелл сои определяют способность одних сортов выступать в роли лучших материнских родителей, а других – быть лучшими отцовскими формами. Получены новые полные нуклеотидные последовательности хлоропластного и митохондриального геномов 46 образцов культурной сои с применением метода секвенирования нового поколения (NGS) на платформе Illumina. Выполнено комплексное биоинформатическое сравнительное исследование внутривидовой изменчивости геномов органелл у 46 сортов сои разнообразного географического происхождения. Выявлены полиморфные локусы геномов. Данные об изменчивости ДНК верифицированы секвенированием по Сэнгеру. Спектр изменчивости органельных ДНК, исследованных методом полногеномного секвенирования сортов сои, представлен тремя гаплотипами хлоропластной ДНК (С1–С3) и пятью гаплотипами митохондриальной ДНК (М1–М5). Обнаружен сравнительно низкий уровень внутривидовой изменчивости гено-мов органелл у G. max. Хлоропластный геном сои обладал меньшим уровнем изменчивости последовательности, чем митохондриальный. Разработан набор ДНК-маркеров к полиморфным локусам геномов органелл, позволяющий дифференцировать сорта сои на плазматипы. Методом ПЦР с последующим секвенированием по Сэнгеру дополнительно изучено 90 образцов сои из коллекции. Низкий уровень внутривидовой изменчивости геномов органелл у G. max подтвержден на расширенной группе образцов. Большая часть коллекции была представлена тремя плазматипами – С1/М1, С2/М2 и С1/М3. 46 полных последовательностей хлоропластной ДНК помещено в NCBI GenBank. Гипотеза влияния ДНК органелл на комбинационную способность различных сортов в настоящее время не подтверждена. Требуются более детальное изучение механизмов ядерно-цитоплазматического взаимодействия, поиск ядерных маркеров, влияющих на экспрессию цитоплазматических генов.

## Introduction

The study of the organization and function of cellular organelle genomes is a significant area of modern plant genetics. Over the past decades, numerous studies have been dedicated to this topic, addressing issues of taxonomy, phylogeny, nuclear-cytoplasmic coadaptation, and genomic interactions (Gualberto, Newton, 2017; Johnston, 2019; Tsunewaki et al., 2019). The organelle genomes of major agricultural crops are actively studied worldwide (Siniauskaya et al., 2020; Hu et al., 2022; Yue et al., 2023).

Soybean (Glycine max (L.) Merr) has been grown in Belarus for many years. In the 1980s, a national school of soybean genetics and breeding originated at the Laboratory of Cytoplasmic Inheritance of the Institute of Genetics and Cytology of the National Academy of Sciences of Belarus. The main focus of soybean breeding in Belarus is the development of early and ultra-early maturing cultivars with sufficiently high yields and seed protein content

The selection of initial material for hybridization is a key element in the breeding of any crop. Cultivars are known to differ in their ability to produce new hybrids and varieties. Therefore, pedigree analysis and the search for such cultivars are necessary. Based on a study of the pedigrees of hybrids and cultivars developed by other researchers and on our own data, we have identified soybean cultivars that are the most promising as either maternal or paternal parents and are most frequently used in crossbreeding. Given the strictly maternal inheritance of organelles in soybeans, it can be assumed that the observed differentiation of cultivars in their ability to produce successful new gene combinations in hybrids is determined by nuclear-cytoplasmic interactions, possibly due to the organization and expression of organelle genomes.

The availability of collections of original plant breeding material with wide variation in a range of economically valuable traits is a necessary prerequisite for the development of new promising varieties. The study of the phenotypic and genetic variability of collections is one of the fundamental areas of modern breeding, including marker-assisted selection.

The first molecular genetic studies on the variability of the organelle genomes of cultivated and wild soybeans were conducted in the 1980s using restriction fragment length polymorphism (RFLP) analysis. The classification of cultivated and wild soybean lines into three chloroplast DNA (cpDNA) haplotypes and five mitochondrial DNA (mtDNA) haplotypes was the standard at that time (Shoemaker et al., 1986). The RFLP method was used to assess the diversity of organelle genomes in wild soybean (Glycine soja) lines (Abe et al., 1999) and soybean varieties cultivated in China (Shimamoto et al., 1998).

The possibility of a more detailed study of the diversity of plant organelle genomes, including those of soybean, using variation in the length of cpDNA microsatellite repeats (SSRs) was demonstrated by W. Powell et al. (1995). This approach enabled the expansion of knowledge on soybean cpDNA variability and the acquisition of qualitatively new data. For example, D. Xu et al. (2002) analyzed six chloroplast microsatellites and identified 52 haplotypes of G. soja and eight haplotypes of cultivated soybean, with 75 % of G. max lines belonging to a single most common haplotype.

The analysis of the variability of soybean chloroplast genomes began at the Institute of Genetics and Cytology of the National Academy of Sciences of Belarus in the 2000s. As a result of the PCR-RFLP study of the collection of 60 varieties available in the Laboratory of Cytoplasmic Inheritance at that time, three plasmatypes were identified, depending on the absence or presence of EcoRI and ClaI restriction enzyme recognition sites in cultivars’ cpDNA. Most varieties (out of more than 60 studied) had an additional ClaI site, three lines had an additional EcoRI site, and the cpDNA of two lines lacked ClaI and EcoRI restriction sites (Siniauskaya et al., 2004). Microsatellite repeats of cpDNA were also studied, and the diversity of soybean varieties collections of various origins was assessed (Aksyonova et al., 2007).

The results of the analysis of the molecular diversity of organelle genomes obtained by a number of author groups have consistently demonstrated that wild soybean has a higher level of cytoplasmic variability than its cultivated relative: the majority of G. max varieties were assigned to one or two major haplotypes (Shimamoto, 2001; Xu et al., 2002). Varieties of Chinese origin were more genetically diverse than those grown in North America and Europe (Yue et al., 2023). The low level of polymorphism of organelle genomes in soybean can be explained by many factors: uniparental inheritance of organelles in soybean through the maternal line, which excludes the possibility of recombination of genomes from two parents; the peculiarities of the propagation of cultivated soybean, for example, strict self-pollination.

The study of the structural features of organelle genomes provides information that can be used to consider (and speculate on) possible microevolutionary changes within the genus Glycine. The generally accepted theory is that the cultivated soybean originated monophyletically from the ancestral form G. soja. This hypothesis was put forward based on studies of nuclear markers, whole-genome sequencing and nuclear SNP panels (Jeong et al., 2019). However, several studies (Xu et al., 2002; Fang et al., 2016) proposed a theory of repeated processes of soybean domestication. The origin of modern cultivated varieties from multiple primordial maternal lines is indicated by features of the chloroplast and mitochondrial genomes; in particular, in most varieties, organelle genomes belong to one of two main plasmatypes. C. Fang et al. divided all cpDNA haplotypes into two groups and suggested that several maternal lines of wild soybean were involved in the domestication of soybean, giving rise to two groups of plasmatypes in modern varieties (Fang et al., 2016).

The development of next-generation sequencing (NGS) methods has provided fundamentally new information on the organization of plant organelle genomes, including those of soybean, their sequences and variability. In 2023, Y. Yue et al. analyzed NGS data from over 2,000 soybean lines from publicly available databases and found that 69.2 % of all cultivated soybean varieties belong to a single plasmatype, CT1/ MT1, while 18.1 % belong to the CT2/MT2 plasmatype, which is consistent with data from earlier studies (Yue et al., 2023).

We previously successfully applied whole-genome sequencing (Illumina platform) to study and assess the diversity of cp and mtDNA in barley (genus Hordeum) (Siniauskaya et al., 2020); an approach to analyzing the results of wholegenome sequencing of organelle DNA was developed. In this study, we aimed to use NGS (Illumina) to obtain new data on whole-genome organelle DNA sequences and assess their level of variability using samples from the collection of early and ultra-early maturing soybean varieties of the Laboratory of Cytoplasmic Inheritance of the Institute of Genetics and Cytology of the National Academy of Sciences of Belarus.

## Materials and methods

DNA extraction. The study material consisted of organelle and total DNA samples of 46 early maturing soybean varieties from the collection of the Institute of Genetics and Cytology, including varieties of Belarusian breeding (the full list is given in the Supplementary Material)1. The selected varieties were successful maternal or paternal forms according to the authors’ long-term data (unpublished data).

Supplementary Materials are available in the online version of the paper: https://vavilov.elpub.ru/jour/manager/files/Suppl_Alex_Engl_30_2.pdf


To isolate organelles using a modification of the method of S.O. Triboush et al. (1998), the first young leaves of 7- to 10-day-old soybean seedlings were used. The isolated organelles were lysed, and the organelle DNA was purified with phenol-chloroform (standard protocol). The quality of the organelle DNA preparation was evaluated by RFLP analysis in 0.8 % agarose gel according to the method of S.O. Triboush et al. (1998).

Total DNA was isolated using phenol-chloroform extraction from the leaves of plants grown in greenhouses or from the first leaves of 7-day etiolated soybean seedlings (standard protocol).

Next-generation sequencing. The organelle DNA was analyzed by paired-end sequencing on an Illumina MiSeq platform using the MiSeq Reagent Kit v3 (600 cycles) (Illumina Inc., USA) following the manufacturer’s recommendations. To prepare the DNA library, the Illumina DNA Prep, (M) Tagmentation (24 Samples, IPB) and Nextera XT Index Kit v2 Set A (96 indexes, 384 samples) (Illumina Inc., USA) kits were used following with the manufacturer’s recommendations.

NGS data analysis. Whole-genome sequencing data were processed according to a previously developed algorithm (Yermakovich et al., 2020), which included: alignment of reads to reference sequences of the chloroplast and mitochondrial genomes, conversion to bam files and their sorting, generation of VCF files and their filtering. Read alignment files (bam) were visualized using Unipro Ugene and IGV. The chloroplast genome assemblies of the Bragg cultivar (GenBank accession number MW357264) and the mitochondrial genome of the Aiganhuang cultivar (NC020455) were used as reference genomes.

NGS data verification. A set of primers was designed for polymorphic loci of organelle genomes identified after analysis of whole-genome sequencing data to verify sites of variability using Sanger sequencing.

Polymorphic DNA regions were amplified separately in 15 μl of a reaction mixture containing 30–40 ng of sample DNA, 7.5 μl of 2x ArtMix reagent mixture (ArtBioTech LLC, Republic of Belarus), and 1 μl of the corresponding primers (5 pmol/μl) (Primetech ALC, Republic of Belarus). PCR was performed on a C1000 amplifier (Bio-Rad Laboratories, Inc., USA) according to the following protocol: 5 minutes at 95 °C, then 30 cycles, each of which included denaturation at 95 °C for 30 seconds, primer annealing for 30 seconds, and elongation at 72 °C for 25–80 seconds, followed by a final elongation at 72 °C for 5 minutes. The PCR product was identified in 1.5 % agarose gel.

Sanger sequencing of the studied samples was performed on an Applied Biosystems 3500 Genetic Analyzer (Thermo Fisher Scientific Inc., USA) using the BrilliantDye Terminator v3.1 Cycle Sequencing Kit (NimaGen B.V., Netherlands), according to the manufacturer’s recommendations. Sequencing results were analyzed using Chromas and FinchTV software by comparing the obtained DNA sequences with the G. max reference genome (Bragg and Aiganhuang cultivars) from NCBI GenBank.

Phylogenetic analysis was performed by creating a presence/ absence matrix based on cp and mtDNA polymorphisms using Mesquite program (http://www.mesquiteproject.org), calculating a distance matrix and constructing a dendrogram in the phylip program (Felsenstein, 1989) based on the neighborjoining algorithm. Dendrogram visualization was performed using the Iroki online service (Moore, 2020).

Chloroplast genomes assembly. Complete cpDNA sequences of all 46 cultivars used for the study were obtained by manually editing the Bragg chloroplast genome reference sequence to account for the identified polymorphisms.

## Results and discussion

**Chloroplast genome variability**

Nine polymorphic sites were identified in cpDNA. Intervarietal variability in soybean cpDNA was represented by five single-nucleotide polymorphisms (SNPs) and four microsatellite repeat regions. Four out of the five identified SNPs were located in the coding regions of the atpB, rps4, accD and rps3 genes; all were synonymous. Almost all of the detected SNPs and repeats were located in the large single-copy region (LSC), and only one SNP was in the small single-copy region (SSC).

In a number of studies searching for interspecific and intergeneric variability in the chloroplast genomes of other crops, “hot spots” of cpDNA variability have been identified, such as ccsA-ndhD, trnH-psbA, ndhG-ndhI, rps18-rpl20, rps15-ycf1, psbZ-trnG-trnS, trnK-rps16, trnD-trnY, trnW-trnP, rpl33- rps18, petG-trnW, atpB-rbcL and rpl32-trnL (Iram et al., 2019; Mehmood et al., 2020). We did not find any differences in these regions, except for the SSR locus in the intergenic sequence atpB-rbcL, apparently due to the taxonomic similarity of the soybean varieties studied.

Of the four polymorphic cpDNA microsatellite loci identified, the SSR locus at position 6,967 (T11–T10) corresponds to the previously described gmcp4 locus (Xu et al., 2002). The remaining microsatellites are newly described and can be used to identify genetic variability in the soybean chloroplast genome.

A single nucleotide substitution at position 82,035 in the coding sequence of the rps3 gene affects the ClaI restriction site and was previously described as a polymorphic locus by which soybean varieties are differentiated into two types using RFLP (Kanazawa et al., 1998). This same SNP in the rps3 gene was included by Y. Yue et al. as a representative locus (position 82,028 in the plastid genome of Zhonghuang 13). This locus, according to the classification of Y. Yue et al., divides all varieties into two groups: the first group (1,848 lines) is characterized by the presence of an alternative allele at position 82,028 (haplotypes CT1, CT4, CT7, CT22, CT28, CT33 and CT44), the second group has a reference allele (the remaining 732 lines) (Yue et al., 2023).

As a result of our study, nucleotide sequences of chloroplast genomes of 46 soybean cultivars were obtained and published in NCBI GenBank under accession numbers OQ148707– OQ148730 and OR834463–OR834484.

**Mitochondrial genome variability**

Comparative analysis of mitochondrial genome sequences obtained from whole-genome sequencing of 46 soybean varieties revealed 15 polymorphic loci: three SNPs in noncoding intergenic regions, eight polymorphic SSR loci, three INDELs (insertion/deletion) and one inversion. Only two out of the 15 loci were located in putative coding regions: one mutation in the orf110c microsatellite repeat (position 294,729 bp in the reference genome of the Aiganhuang cultivar), and an inversion in orf160b (position 205,470).

We identified variability in the intergenic regions atp6-1- trnK, rps3-orf114a, orf100c-orf136b, trnD-orf114b, orf151- orf261, atp9-trnM and in the nad4 intron of the mitochondrial genome. Similar data were obtained by Y. Yue et al. in 2023, and the polymorphic positions were classified as representative (marker) ones. These are positions 451,199, 284,000, 312,950, 489,992, 256,743, 295,084, and 484,505 in the mtDNA of the Zhonghuang 13 cultivar, which correspond to the regions mentioned above. These polymorphic loci allow differentiation of the MT1 and MT2 haplotypes according to the classification of Y. Yue et al. (2023).

To confirm the detected loci in the cp and mt genomic DNA, a set of primers targeting sites of genomic variation was designed, and a Sanger sequencing study of these polymorphic loci was conducted in 46 accessions. The primers were designed to study the most important marker points of genomic variation used to differentiate individual cp and mtDNA haplotypes in soybean varieties: positions 6,967, 75,657, and 116,598 of the chloroplast genome, and positions 100,092, 197,000, 205,470, and 248,977 of the mitochondrial genome. Primer combinations were also developed to study the most ambiguous or difficult-to-interpret regions of the genome when analyzing bam and VCF files: SSR locus 51,525 of cpDNA and INDELs at positions 158,807 and 321,983 of mtDNA. All differences identified between the varieties were confirmed.

**Genetic diversity of soybean organelle genomes**

Based on chloroplast genome variability, the studied varieties were divided into three haplotypes: 40 varieties (87 %) were assigned to haplotype I, four varieties (Kitrossa, Lyubasha, Oressa, and Voronezhskaya 31), to haplotype II, and the Legenda and Schara varieties, to haplotype III. The identified haplotypes were designated C1–C3.

Based on 16 polymorphic loci in mitochondrial DNA, five haplotypes were identified, designated M1–M5. Ten INDELs and four SNPs were present in the Lyubasha, Kitrossa, Oressa, and Voronezhskaya 31 varieties (haplotype M2), one INDEL was found in the related varieties Vasilisa, Amazonka, McCall, Ptsich, and Sahara (haplotype M3). Cultivars Optimus (M5) and Zlata (M4) also differ from other varieties by an inversion and one INDEL, respectively. Most varieties (76 %) belong to the M1 haplotype. Tables 1 and 2 present the differences identified in the organelle genomes and their distribution among the various cp and mtDNA haplotypes.

**Table 1.**
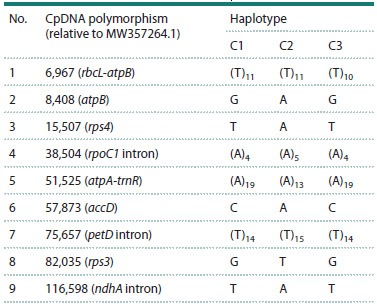
Polymorphic loci of soybean chloroplast DNA and haplotypes identified on their basis

**Table 2.**
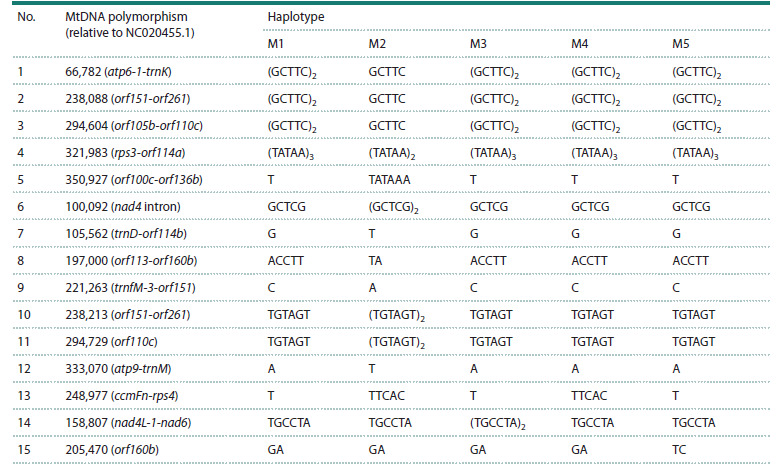
Polymorphic loci of soybean mitochondrial DNA and haplotypes identified on their basis

Based on our data, an analysis of any of the soybean varieties studied by microsatellite repeats No. 1 and 5 allow to distinguish between cpDNA types C1, C2, and C3. These repeats can be recommended for use in studies of genetic diversity in various soybean collections using the chloroplast genome without the use of other cpDNA markers.

Additionally, Sanger sequencing was performed for 90 varieties from the collection using primers differentiating the corresponding haplotypes of cp and mtDNA. Based on the summarized results of whole-genome sequencing and Sanger sequencing, six organelle DNA plasmatypes are identified among the studied varieties of our collection (the so-called northern ecotype): C1/M1, C2/M2, C1/M3, C1/M4, C1/M5 and C3/M1. 65.4 % of soybean varieties (89 out of 136 studied) belong to the C1/M1 plasmatype. 20 (14.7 %) and 23 (16.9 %) varieties have the C2/M2 and C1/M3 plasmatypes, respectively. Three out of 136 varieties (2.2 %) belong to the C1/M4 plasmatype. Plasmatypes C1/M5 and C3/M1 are the rarest, comprising one (0.7 %) and two (1.4 %) varieties, respectively (Table 3).

**Table 3.**
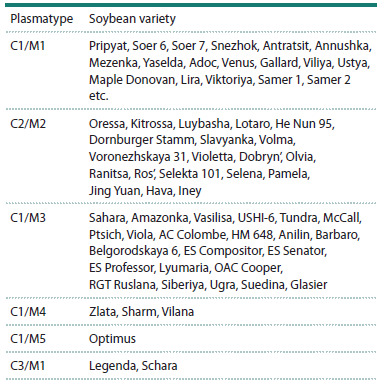
Differentiation of soybean varieties into plasmatypes based on NGS and Sanger sequencing results

Based on the phylogenetic analysis, the identified plasmatypes are divided into two clades. One of these clades consists of plasmatypes C1/M1, C1/M3, C1/M4, C1/M5, and C3/M1, which are most similar to each other in terms of organelle genome sequences. Haplotype C2/M2 constitutes the second clade and has the greatest number of differences from haplotype C1/M1 – eight cpDNA polymorphisms and 13 mtDNA polymorphisms (see the Figure).

**Fig. 1.**
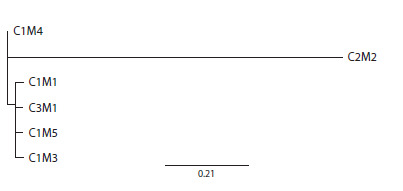
Dendrogram of phylogenetic relationships in the studied collection of soybean varieties

Considering that the vast majority of soybean varieties we studied have plasmatype C1/M1, it can be considered the main one in the collection. Plasmatypes C1/M3, C1/M4, C1/M5 and C3/M1 are most likely derived from it, since they differ from C1/M1 by only one polymorphic loci in the chloroplast (C3) or mitochondrial genomes (M2–M5).

The C1/M1 and C2/M2 plasmatypes identified by us are consistent with the CT1/MT1 and CT2/MT2 plasmatypes discovered by Y. Yue et al. in 2023. According to their study, these plasmatypes are the most common among cultivated soybean varieties – 69.2 and 18.1 %, respectively (Yue et al., 2023). In our study, we confirmed that the CT1/MT1 plasmatype (C1/M1 according to our classification) is also prevalent among Belarusian varieties. We also identified rare differences not previously described by other researchers. Based on these differences, we identified five additional plasmatypes, of which C1/M3 is more common than C2/M2 in our collection of varieties. This is presumably explained by the geographic and ecological characteristics of Belarus and the selection of varieties that, based on a number of other traits, are most suitable for cultivation in Belarus.

Unfortunately, the fundamental hypothesis of our study regarding the possible organelle genome features that differentiate the best maternal and paternal soybean varieties has not yet been confirmed, as both varieties have similar cp and mtDNA haplotypes. To clarify this issue, a more in-depth study of the subtle mechanisms of interaction between the nucleus and cytoplasm is required.

## Conclusion

A comparative analysis of NGS data from 46 early maturing soybean cultivars allowed us to identify polymorphic loci in chloroplast and mitochondrial DNA and assess the intraspecific diversity of organelle DNA in soybean varieties of diverse geographic origin.

Nine loci in the chloroplast and 15 loci in the mitochondrial genomes of cultivated soybean were identified. The entire spectrum of soybean organelle DNA variability is represented by three chloroplast DNA haplotypes and five mitochondrial haplotypes. Combining variability across both organelle genomes allowed us to identify six organelle DNA plasmatypes and differentiate varieties in the collection accordingly. Using DNA markers for polymorphic loci in organelle genomes developed based on NGS data analysis, we studied plasmon variability in 90 varieties from the collection of Laboratory of Cytoplasmic Inheritance, characterized by a wide range of origins and sensitivity to day length. Forty-six complete soybean chloroplast DNA sequences have been deposited in NCBI GenBank under the accession numbers OQ148707–OQ148730 and OR834463–OR834484.

Our results and analysis of the pedigrees of the varieties confirm the data of other researchers indicating that the C1 and M1 haplotypes are the most common among varieties cultivated in North America, Europe, and the CIS countries. The C1/M1 plasmatype was also detected with the highest frequency in the collection of soybean accessions we studied. The C2 cpDNA and M2 mtDNA haplotypes are characteristic of modern soybean varieties bred in China. Interestingly, the C2/M2 plasmatype was quite common in our collection among a number of accessions with indigenous Chinese maternal forms in their pedigree.

The identification of six plasmatypes indicates a low level of genetic diversity in the studied collection of soybean varieties. The group of early and ultra-early maturing cultivars we studied is an evolutionarily young and very narrow group of varieties (these are varieties of the northern ecotype). Given the targeted selection of these varieties for day length neutrality, a low level of genetic diversity is quite expected in such a limited group of soybean lines (Rosenzweig et al., 2003).

Identifying sources of cytoplasmic variation and varieties with distinct cpDNA and mtDNA haplotypes, as well as introgression of new genetic material into the breeding process are essential for the productive development of new soybean varieties for northern latitudes.

## Conflict of interest

The authors declare no conflict of interest.
